# Incorporation of Aloe Vera Gel Into Kefir: Impact on Volatile Composition, Rheological, Techno‐Functional, and Microbiological Properties

**DOI:** 10.1111/1750-3841.70292

**Published:** 2025-05-28

**Authors:** İsra Yiğitvar, Ali Adnan Hayaloğlu

**Affiliations:** ^1^ Halal Accreditation Agency Ankara Türkiye; ^2^ Department of Food Engineering İnönü University Malatya Türkiye

**Keywords:** Aloe vera, beverage, kefir, probiotic

## Abstract

**ABSTRACT:**

This study aimed to produce a unique kefir enriched with Aloe vera gel (*Aloe barbadensis* Miller). Kefir samples contain 5%–15% of Aloe vera gel, except for the control sample. Physical, chemical, rheological, microbiological, and sensory properties were determined over a storage period of 21 days. The values for total solids, protein, ash, titratable acidity, pH, and syneresis were between 8.58% and 10.71%, 2.81% and 3.87%, 0.51% and 0.65%, 0.63% and 0.76%, 4.25 and 4.35, and 57.15% and 67.29%, respectively. Rheological and viscosity tests showed that the samples exhibited pseudoplastic behavior, which is typical for non‐Newtonian fluids. The addition of Aloe vera gel led to a decrease in viscosity and flow properties. The values included variations in total phenolic content (101.31–333.61 mg gallic acid equivalent/L [GAE/L]), 2,2′‐azino‐bis(3‐ethylbenzothiazoline‐6‐sulfonic acid) (ABTS) (83.85–99.47 mg Trolox equivalent/L [TE/L]), and 2,2‐diphenyl‐1‐picryhyldrazyl (DPPH) (473.51–615.54 mg TE/L) for the samples containing Aloe vera gel. The addition of Aloe vera gel increased the antioxidant activity (ABTS); however, the total phenolic content decreased. A total of 44 volatile compounds were identified, and the addition of Aloe vera gel resulted in higher levels of 1‐hexanol, benzyl alcohol, and 2‐methylpentanoic acid. The microbiological analysis results were within the prescribed limits for total aerobic mesophilic bacteria, yeasts and molds, lactobacilli, lactococci, and total coliforms. After the completion of the sensory analysis, the samples showed respectable results. Compared with single prebiotics and probiotics, a synbiotic fermented beverage made with Aloe vera gel as a substrate for lactic acid fermentation could have greater health benefits. It is therefore proposed to industrially produce this unique fermented milk product.

**Practical Application:**

Aloe vera (*Aloe barbadensis* Miller) gel‐added kefir is produced in this study as a novel fermented beverage. The physical, chemical, rheological, microbiological, and sensory properties of the samples were determined during storage. The industrial production of this synbiotic fermented milk product is recommended due to its potential impact on health as a stronger probiotic.

## 1 Introduction

Aloe vera (*Aloe barbadensis* Miller) is one of the medicinal and aromatic plants originally from Africa. In addition to various vitamins, minerals, polysaccharides, enzymes, amino acids, proteins, phenols, and organic acids, it has more than 200 bioactive components (Sathyaprabha et al. 2010; Govindammal et al. 2017). Ancient cultures used it in traditional medicine for its antibacterial, antiviral, anticarcinogenic, antioxidant, and anti‐inflammatory properties (Gao et al. 2019).

Kefir is a traditional fermented milk product with a distinct flavor and functional properties. It is a natural and probiotic beverage with strong therapeutic properties, as it contains symbiotic microorganisms, mainly lactic acid bacteria (LAB) (Kıvanc and Yapıcı 2019; Putri et al. 2020). The anticarcinogenic, antimutagenic, antiviral, and antifungal properties of kefir have been explored with in vitro studies (Uruc et al. 2022). As more and more individuals seek to enhance their health through premium‐quality foods, the popularity of plant‐based functional foods is on the rise. For instance, studies have been conducted on the production of kefir enriched with various substances such as fruit juices (Kabakcı et al. 2020), walnut oil (Turek and Wszolek 2022) and coffee flavoring (Vimercati et al. 2020). Aloe vera is expected to bring unique properties to the kefir formulation used in this study, resulting in a fermented beverage with remarkable bioactive and organoleptic properties. Aloe vera gel–enriched kefir could be healthier for consumers than taking probiotics and prebiotics separately. On the basis of the authors’ knowledge, the use of Aloe vera in food is limited to fermented dairy products, for example, yogurt, ayran, and cheese (Govindammal et al. 2017; Rezazadeh‐Bari et al. 2019; El‐Sayed and El‐Sayed 2020). This work aimed to incorporate Aloe vera gel into the kefir as a substrate for LAB fermentation to produce a synbiotic beverage. Therefore, Aloe vera gel is used to produce a novel fermented milk product characterized by the determination of certain rheological, sensory, microbiological, and physicochemical properties of the samples during storage.

## 2 Materials and Methods

### 2.1 Materials

Ultra‐high‐temperature (UHT)‐treated, semi‐skimmed milk and freeze‐dried kefir starter culture (KF1 A 10 U, Maysa, Istanbul, Türkiye) were sourced from local suppliers. The semi‐skimmed UHT milk contains 2.8% protein, 4.7% carbohydrates, and 1.5% fat. Aloe vera leaves that were at least 2.5 years old were harvested and supplied from a local greenhouse in Antalya, Türkiye. A sharp knife was used to properly peel the Aloe vera leaves to extract gel. To have a liquid structure, a mixer was used.

### 2.2 Methods

#### 2.2.1 Kefir Production

The experimental kefir samples were prepared as follows: K1—2‐L milk (control sample) and starter culture; K2—1.9‐L milk, starter culture and 100 mL (5 %) Aloe vera gel; K3—1.8‐L milk, starter culture and 200 mL (10 %) Aloe vera gel; K4—1.7 L milk, starter culture and 300 mL (15%) Aloe vera gel. The concentrations of Aloe vera gel for kefir production were determined in preliminary tests. Similar processes for kefir production were described by Kök‐Taş et al. (2013) and Yılmaz‐Ersan et al. (2018). Semi‐skimmed UHT milk was inoculated at 25°C with 2% kefir starter culture (containing a mixture of *Lactococcus lactis*, *Leuconostoc mesenteroides*, *Levilactobacillus brevis*, and *Saccharomyces cerevisiae*). All samples—with the exception of the control sample—were then treated with Aloe vera gel. The samples were mixed in a mechanical homogenizer (UltraTurrax T25, IKA Werke, Staufen, Germany) at 10,000 rpm for 60 s. Fermentation was carried out at 25°C for 18 h until pH 4.60 and below was reached. The curd was broken up by gently stirring the samples with a spoon. Each batch was prepared in triplicate and stored at 4°C until analysis. The analyses were carried out in triplicate after the 1st, 7th, 14th, and 21st days.

#### 2.2.2 Physicochemical Analyses

The standard AOAC procedures were followed to calculate total solids, ash, protein, and titratable acidity (AOAC 2012). A pH meter was used to determine the pH (Mettler Toledo, S220, USA). A rotational viscometer (Brookfield Viscometer—DV‐II Pro, USA) was used to measure viscosity values at 4°C and 10–100 rpm with spindle number 3. The results were expressed in mPa s. Kefir (5 mL) was centrifuged (Hettich 320 R Tuttlingen, Germany) for 10 min at 2500 × *g* and 4°C to determine syneresis. Subsequently, the syneresis values were calculated using the following formula after the supernatant was weighed (Temiz and Dağyıldız 2017):

(1) $$\mathrm{Syneresis}\;\left(\%\right)=\left(\frac{m_{1}}{m_{2}}\times 100\right)$$

where $m_{1}$ is the weight of sample (g); $m_{2}$ is the weight of supernatant (g).

A colorimeter was used for the color analysis (Minolta, CR‐5, Japan). Red–green intensity (*a* ^*^), yellow–blue intensity (*b* ^*^), and brightness (*L* ^*^) were measured.

The values for total color change (Δ*E*), chroma (*C*), and hue angle were calculated using the following formulas:

(2) $$\Delta E=\sqrt{{\left(L^{*}-L_{0}\right)}^{2}+\left({\left(b^{*}-b_{0}\right)}^{2}\right)+{\left(a^{*}-a_{0}\right)}^{2}}$$

(3) $$C=\sqrt{\left({a^{*}}^{2}+{b^{*}}^{2}\right)}$$

(4) $$\mathrm{hue}=\tan^{-1}\left(\frac{b^{*}}{a^{*}}\right)$$

#### 2.2.3 Determination of Phenolic Components and Antioxidant Activity

The method outlined by Singleton et al. (1999) was used to determine the total phenolic content of the samples containing Aloe vera. The findings are given as mg gallic acid equivalent/L (mg GAE/L).

ABTS*+ (2,2′‐azino‐bis(3‐ethylbenzothiazoline‐6‐sulfonic acid)) Trolox‐equivalent antioxidant and DPPH* (2,2‐diphenyl‐1‐picryhyldrazyl) radical scavenging capacities were used to assess the antioxidant activity of the samples. ABTS and DPPH were measured on the basis of the methods by Xu et al. (2010) and Lucena et al. (2010), respectively. The findings are given as mg Trolox equivalent/L (mg TE/L).

#### 2.2.4 Volatile Component Analysis

The volatile compounds were determined with solid‐phase microextraction (SPME) in Aloe vera gel and kefir samples. 2‐Methyl‐3‐heptanone (300 ppm in methanol) was added as an internal standard to a screw‐capped vial containing 5 mL of the sample. An SPME fiber with DVB/CAR/PDMS (divinylbenzene/carboxen/polydimethylsiloxane; 50/30 µm film thickness, 2 cm length; Supelco, Bellefonte, PA, USA) was used for the extraction. In order to adsorb the volatiles, the fiber was injected into the vial's headspace after the vials had been on the heater at 40°C for 30 min. A DB‐WAX capillary column (60 m × 0.250 mm × 0.25 µm, J&W Scientific, Folsom, CA, USA) was used to separate volatile organic compounds. A Shimadzu (model QP2010, Kyoto, Japan) gas chromatograph mass spectrometry (GC–MS) system was used to define the volatile organic compounds. The peaks were identified by comparison of MS libraries (NIST and WILEY) and authentic standards. The amounts of volatile compounds were calculated on the basis of the peak areas from the internal standard and given as µg/g (Korkmaz et al. 2020).

#### 2.2.5 Determination of Peptide Profiles

An RP‐HPLC (Shimadzu LC 20 AD Prominence HPLC, Kyoto, Japan) was used to determine the peptide profiles of the kefir samples. The separation was performed using a Zorbax 300 Å SB C18 (4.6 × 250 mm^2^, USA) column. Peptide extraction was performed following the procedure described by Ebner et al. (2015). For elution, 40 µL of the extract was injected into the system. Elution was carried out at 214 nm while maintaining a column oven temperature of 35°C. All chromatographic conditions were followed as described by Şanlı et al. (2018). The following solvents were used: (A) 0.1% (v/v) trifluoroacetic acid (TFA, Sigma, St. Louis, USA) was prepared in HPLC‐grade deionized water (Milli‐Q system, Waters Corp., Molsheim, France), and (B) 0.1% (v/v) TFA was prepared in acetonitrile (Fluka ≥99.5%, Sigma‐Aldrich Chemie, CH‐9471 Buchs, USA) with a flow rate of 0.75 mL/min. The chromatographic conditions of peptides were the same as in Şanlı et al. (2018).

#### 2.2.6 Rheological Evaluation

A rheometer (Anton Paar MCR301, Germany) was used to measure the kefirs’ rheological properties. A PP25 parallel plate was used; the gap value and space between the plates were 1 and 30 mm, respectively. The frequency was adjusted between 0.1 and 10 Hz, and the strain was 1%. Kefir of 1 mL was placed between the parallel plates at 20°C. The data were determined using Anton Paar software. The flow behavior of the kefir samples was determined by measuring the shear rate (1/s) and the shear stress (Pa). Dynamic measurements and gel properties of the kefir were determined by measuring the frequency values (Hz) against the elastic (*G*′) and viscous (*G*″) moduli (Bensmira et al. 2010).

#### 2.2.7 Microbiological Analysis

Serial dilutions prepared from 1 mL kefir and 0.85% sodium chloride solution (w/v) were used to count the microorganisms. Then 0.5 mL of the dilution for each microorganism was added to the corresponding medium. Total aerobic mesophilic bacteria (TAMB) were incubated for 2 days at 37°C on plate count agar (LAB M Limited, UK). Yeasts and molds were incubated for 4 days at 25°C on potato dextrose agar (LAB M Limited, UK). Lactobacilli were incubated for 48 h at 37°C on MRS agar prepared from MRS broth (LAB M Limited, UK) and agar (Liofilchem, Italy). Lactococci were cultured for 48 h at 37°C on M17 agar (Sigma‐Aldrich, Germany). Coliforms were incubated for 2 days at 37°C on Violet Red Bile Agar (LAB M Limited, UK). *Escherichia coli* was incubated for 48 h at 44°C on eosin methylene blue agar (Sigma‐Aldrich, Germany). The number of viable microorganisms in kefir was expressed as log_10_ CFU/mL (Isık et al. 2023).

#### 2.2.8 Sensory Evaluation

A sensory examination was carried out in accordance with the Turkish standard (TS 5546, 2009). There were nine panelists (five female and four male) who were trained in sensory analysis, academic staff, and postgraduate students of Inönü University Department of Food Engineering, aged 24–44 years. Color and appearance, milky odor, fermented odor, herbal odor, flavor, sour taste, bitter taste, milky taste, texture, acidity, and overall acceptability were evaluated. Samples were randomly assigned codes and displayed in a regulated setting with ambient temperature, consistent lighting, minimum noise, and adequate air circulation. Panelists rated each attribute on a scale of 1–10: 1 for “I do not like” and 10 for “I like very much.” When selecting the test subjects, attention was paid to their state of health to ensure that they were accustomed to the evaluation procedure and that there were no conditions that could impair their sensory impressions. To lessen the number of residual flavors, each participant was instructed to rinse their mouth with water after tasting one sample at a time. The aim of this method was to increase the precision and dependability of the collected sensory data that was gathered.

#### 2.2.9 Statistical Analysis

The SPSS 16.0 (SPSS Inc., USA) package program was used to evaluate the experimental data at a significance level of *p* < 0.05 using Duncan's multiple comparison tests and one‐way ANOVA. To interpret the results for the different treatments, the effects of formulation (*F*, the amount of Aloe vera gel), storage (*S*), and interactions *S* × *F* on the results were determined by a factorial experimental design. The data from the GC–MS analysis of the volatile components were assessed by principal component analysis (PCA) using the same package program.

## 3 Results and Discussion

### 3.1 Physicochemical Properties

The average total solids, protein and ash content, and pH of Aloe vera gels were 1.03% ± 0.11%, 0.20% ± 0.07% and 0.22% ± 0.00%, and 4.54 ± 0.02, respectively. The result of the current study concurs with previous studies by Miranda et al. (2009) and Vega‐Gálvez et al. (2011). The total solids, total protein and ash contents, and pH of Aloe vera pulp were found to be 1.50% ± 0.1%, 0.17% ± 0.01% and 0.07% ± 0.1%, and 4.16 ± 0.03, respectively. The total solid content of the kefirs is shown in Table 1 and ranged from 8.58% to 10.71%. The total solids decreased incorporating Aloe vera gel during storage (*p* < 0.05). The total solid content was reported to be 9.56%–19.91% for kefir produced with electro‐activated whey (Aidarbekova and Aider 2022) and 7.93%–8.15% for kefir produced with microbial transglutaminase (Temiz and Dağyıldız 2017). The total protein values of kefir samples varied between 2.81% and 3.87% (Table 1). The results are in accordance with the regulations (Anonymous 2022) for kefir in Türkiye; it should be at least 2.70%. Total protein values decreased incorporating Aloe vera gel, as seen in the total solid content; the results were statistically significant (*p* < 0.05) on the 21st day of storage. The use of different formulations in kefir production significantly changed the total protein values (*p* < 0.01). Turek and Wszolek (2022; 3.51%–3.54% for kefir produced with walnut oil) and Kök‐Taş et al. (2013; 3.09%–3.48% for kefir produced with different fermentation parameters) found almost the same values for total protein. The ash content of the kefir samples ranged from 0.51% to 0.65% (Table 1). The use of different formulas in kefir production resulted in significant differences in total ash content (*p* < 0.05). It was reported that the total ash content ranged between 0.61% and 1.84% for coffee‐flavored kefir (Vimercati et al. 2020) and 0.28% and 0.64% for apricot kernel extract kefir (Uruc et al. 2022).

**TABLE 1:** Some physical and chemical properties of kefir samples containing 0% (K1), 5% (K2), 10% (K3), and 15% (K4) levels of Aloe vera.

	Storage (day)	Sample			
		K1	K2	K3	K4
Total solids (%)	1	10.65 ± 0.49^b,A^	10.17 ± 0.45^b,A^	9.66 ± 0.43^ab,A^	8.96 ± 0.44^a,A^
	7	10.31 ± 0.21^b,A^	9.97 ± 0.33^b,A^	9.64 ± 0.45^b,A^	8.82 ± 0.27^a,A^
	14	10.71 ± 0.62^a,A^	10.37 ± 0.24^a,A^	9.45 ± 0.28^a,A^	9.21 ± 1.01^a,A^
	21	10.64 ± 0.85^a,A^	10.19 ± 0.89^a,A^	9.15 ± 0.40^a,A^	8.58 ± 1.18^a,A^
Total protein (%)	1	3.58 ± 0.20^a,A^	3.56 ± 0.36^a,A^	3.47 ± 0.46^a,A^	3.21 ± 0.81^a,A^
	7	3.87 ± 0.22^a,A^	3.32 ± 0.04^a,A^	3.35 ± 0.33^a,A^	3.18 ± 0.48^a,A^
	14	3.53 ± 0.15^a,A^	3.03 ± 0.44^a,A^	3.07 ± 0.27^a,A^	2.96 ± 0.19^a,A^
	21	3.57 ± 0.35^c,A^	3.44 ± 0.12^bc,A^	2.81 ± 0.21^a,A^	2.95 ± 0.06^ab,A^
Total ash (%)	1	0.57 ± 0.06^a,A^	0.57 ± 0.04^a,A^	0.56 ± 0.05^a,A^	0.51 ± 0.06^a,A^
	7	0.60 ± 0.06^a,A^	0.60 ± 0.05^a,A^	0.58 ± 0.03^a,A^	0.56 ± 0.05^a,A^
	14	0.65 ± 0.03^a,A^	0.62 ± 0.04^a,A^	0.59 ± 0.04^a,A^	0.57 ± 0.03^a,A^
	21	0.62 ± 0.04^a,A^	0.58 ± 0.03^a,A^	0.57 ± 0.04^a,A^	0.55 ± 0.05^a,A^
Titration acidity (% lactic acid)	1	0.71 ± 0.01^a,A^	0.69 ± 0.02^a,A^	0.70 ± 0.02^a,A^	0.68 ± 0.04^a,A^
	7	0.76 ± 0.02^c,A^	0.71 ± 0.01^b,A^	0.70 ± 0.02^ab,A^	0.66 ± 0.00^a,A^
	14	0.73 ± 0.03^b,A^	0.71 ± 0.01^b,A^	0.69 ± 0.01^ab,A^	0.66 ± 0.03^a,A^
	21	0.72 ± 0.01^b,A^	0.72 ± 0.04^b,A^	0.66 ± 0.02^ab,A^	0.63 ± 0.02^a,A^
pH	1	4.35 ± 0.00^a,A^	4.33 ± 0.01^a,A^	4.34 ± 0.02^a,B^	4.30 ± 0.04^a,A^
	7	4.33 ± 0.03^a,A^	4.29 ± 0.02^a,A^	4.27 ± 0.04^a,AB^	4.27 ± 0.03^a,A^
	14	4.30 ± 0.03^a,A^	4.29 ± 0.02^a,A^	4.26 ± 0.03^a,AB^	4.28 ± 0.04^a,A^
	21	4.28 ± 0.03^a,A^	4.28 ± 0.04^a,A^	4.25 ± 0.03^a,A^	4.25 ± 0.02^a,A^
Syneresis (%)	1	57.78 ± 1.75^a,A^	61.28 ± 1.29^ab,A^	62.57 ± 2.36^ab,A^	65.79 ± 1.58^b,A^
	7	57.74 ± 2.13^a,A^	64.95 ± 0.95^a,A^	63.94 ± 2.91^a,A^	67.29 ± 1.54^a,A^
	14	57.15 ± 1.79^a,A^	61.14 ± 0.91^a,A^	64.25 ± 0.74^a,A^	63.59 ± 2.68^a,A^
	21	57.83 ± 1.58^a,A^	58.49 ± 1.54^a,A^	65.08 ± 0.68^b,A^	66.84 ± 1.84^b,A^

*Note*: Values indicated by different letters (a–c) in the same row are different from each other at *p* < 0.05 level. Values indicated by different letters (A,B) in the same column are different from each other at *p* < 0.05 level.

The protein, ash, and total solid content of the kefir samples were significantly impacted by the addition of Aloe vera gel. The constituents decreased with increasing amounts of Aloe vera gel added, due to the change in mass balance as the Aloe vera gel was mixed and added to kefir in a liquid state. These findings align with those reported in El‐Sayed and El‐Sayed's earlier study (2020).

Table 1 demonstrates the titratable acidity values of kefir, which varied from 0.63% to 0.76%. The findings are consistent with the minimum value of 0.60% required by the Turkish Food Codex for Fermented Dairy Products (Anonymous 2022). Except for the results on the first day of storage, the titratable acidity values of kefir decreased with the addition of Aloe vera gel (*p* < 0.05). This is due to post‐acidification by the activities of LAB and was previously described by Azari‐Anpar et al. (2017). The use of different formulas in kefir production considerably changed the titratable acidity values (*p* < 0.001). Titratable acidity values have been reported between 0.64% and 1.12% for kefir samples during 12 weeks of storage (Kabakcı et al. 2020) and 0.71% and 1.04% for kefir produced with additional cultures during 28 days of storage (Şanlı et al. 2018).

Table 1 shows that the kefirs’ pH ranged from 4.25 to 4.35. The Aloe vera gel addition and storage had no statistically significant effect on the pH values of the samples (*p* > 0.05). In other studies in the literature, the pH values have been changed between 4.01 and 4.58 for kefir samples during 12 weeks of storage (Kabakcı et al. 2020) and 4.10 and 4.34 for kefir made with goat milk (Putri et al. 2020).

The syneresis values of kefirs are shown in Table 1 (57.15%–67.29%). The syneresis values were increased with the inclusion of Aloe vera gel on the 21st day of storage (*p* < 0.05). It is known that there is a positive correlation between syneresis and acidity in fermented dairy products (Fox et al. 2000; Michael et al. 2010). These results could be due to the high titration acidity produced by the microorganisms of the starter culture, and the presence of Aloe vera gel decreased the colloidal stability of casein micelles, as in the study of Azari‐Anpar et al. (2017). The use of different formulations in kefir production changed the syneresis values (*p* < 0.001). Bensmira and Jiang (2012) reported that the syneresis values of kefir increased with the addition of peanut milk/peanut fat milk. Temiz and Dağyıldız (2017) found that the syneresis values of kefir produced with microbial transglutaminase changed between 52.35% and 74.10%.

**FIGURE 1:**
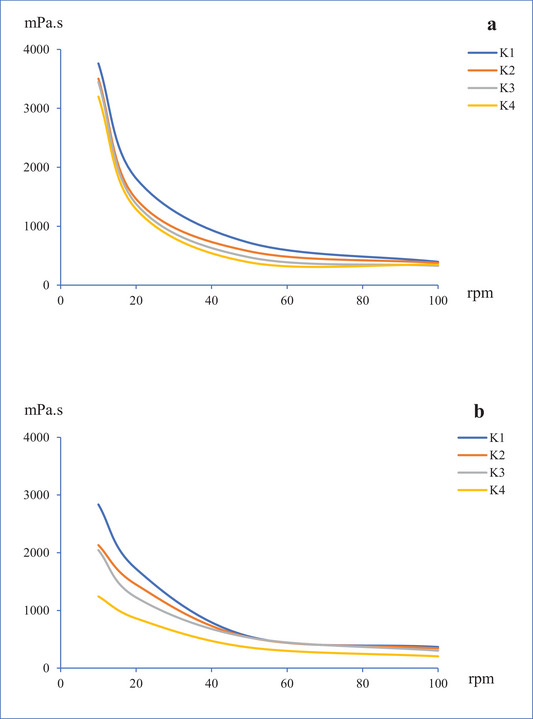
Viscosity values of kefir samples containing 0% (K1), 5% (K2), 10% (K3), and 15% (K4) levels of Aloe vera after 1st (a) and 21st (b) days of storage.

The viscosity values of the kefirs were measured on the 1st and 21st days of storage, as illustrated in Figure 1. The viscosity values (mPa s) decreased with increasing speed (rpm). It has been concluded that the samples exhibited pseudoplastic flow as a characteristic of non‐Newtonian behavior. All samples demonstrated a decrease in viscosity values throughout storage, with the control sample (K1) showing the highest viscosity values. A decrease in viscosity is generally caused by two factors: first, an increase in shear rate, which is associated with a decrease in the alignment of the molecules; and second, the presence of Aloe vera gel in the milk, which contributes to the formation of a fragile coagulum. The colloidal stability of milk is affected by the high content of reactive polyphenols in Aloe vera gel (Nejatzadeh‐Barandozi 2013). Due to the higher casein stability of these active ingredients, an increase in the coagulation period of milk throughout incubation is undesirable for fermented milk products (Fox 1993). The production of yogurt containing Aloe vera gel was investigated, and a considerable decrease in viscosity values was observed with the increase of Aloe vera gel content in the samples (Azari‐Anpar et al. 2017). Another study investigated the effect of different fermentation conditions on the quality characteristics of kefir and found that the viscosity values of the samples decreased over time (Kök‐Taş et al. 2013).

The average *L* ^*^, *a* ^*^, and *b* ^*^ values of Aloe vera gel were 90.47 ± 2.93, (−0.47 ± 0.06), and 11.75 ± 0.37, respectively. The average Δ*E*, hue, and *C* values of Aloe vera gel were calculated as 10.10 ± 0.17, (−86.66) ± 0.64, and 16.01 ± 0.52, respectively. Table 2 demonstrates the color values of the kefirs. The L^*^, a^*^, and b^*^ values of kefir varied between 21.05 and 25.93, 2.67 and 5.11, and 30.77 and 34.05, respectively. The Δ*E*, hue, and C values of kefir ranged from 0.17 to 3.60, 81.03 to 85.39, and 30.83 to 34.83, respectively. The Δ*E* values of samples K1 and K2 changed with storage (*p* < 0.05). The use of different formulas in kefir production significantly changed the a^*^ and hue values (*p* < 0.001). Vimercati et al. (2020) found that the *L* ^*^, hue, and C values of coffee‐flavored kefir ranged from 57.65 to 83.21, 74.18 to 93.87, and 14.68 to 31.85, respectively. In another study, the *L* ^*^, C^*^, and hue values of kefir produced with electro‐activated whey ranged from 72.89 to 84.45, 2.76 to 14.31, and 90.4 to 158.9, respectively (Aidarbekova and Aider 2022). The current study's physicochemical characteristics were mostly in line with those of the previously cited investigations.

**TABLE 2:** Color properties of kefir samples containing 0% (K1), 5% (K2), 10% (K3), and 15% (K4) levels of Aloe vera.

		Sample			
	Storage (day)	K1	K2	K3	K4
L^*^	1	21.05 ± 0.61^a,A^	21.90 ± 0.87^a,A^	23.17 ± 1.11^a,A^	23.56 ± 1.41^a,A^
	7	22.36 ± 2.23^a,A^	22.57 ± 1.00^a,A^	23.39 ± 1.10^a,A^	24.20 ± 2.52^a,A^
	14	23.51 ± 1.40^a,A^	23.45 ± 3.09^a,A^	22.90 ± 2.35^a,A^	23.28 ± 1.77^a,A^
	21	22.32 ± 0.52^a,A^	22.50 ± 0.82^a,A^	25.93 ± 2.26^a,A^	25.50 ± 3.01^a,A^
a^*^	1	5.10 ± 0.34^b,A^	4.60 ± 0.26^ab,A^	3.43 ± 0.02^ab,A^	2.83 ± 0.18^a,A^
	7	4.38 ± 0.39^a,A^	4.40 ± 0.43^a,A^	3.12 ± 0.09^a,A^	2.69 ± 0.06^a,A^
	14	3.86 ± 0.22^a,A^	4.07 ± 0.31^a,A^	3.65 ± 0.37^a,A^	3.30 ± 0.03^a,A^
	21	5.11 ± 0.12^a,A^	4.85 ± 0.03^a,A^	2.71 ± 0.42^a,A^	2.67 ± 0.04^a,A^
b^*^	1	32.70 ± 1.08^a,A^	33.80 ± 0.26^a,A^	34.05 ± 0.84^a,A^	31.82 ± 0.94^a,A^
	7	32.44 ± 1.45^a,A^	33.08 ± 0.71^a,A^	31.98 ± 0.65^a,A^	30.77 ± 1.34^a,A^
	14	33.62 ± 1.25^a,A^	33.01 ± 2.36^a,A^	33.02 ± 1.67^a,A^	32.81 ± 1.04^a,A^
	21	32.18 ± 0.18^a,A^	32.20 ± 0.36^a,A^	32.53 ± 1.41^a,A^	32.11 ± 0.85^a,A^
Δ*E*	1	0.17 ± 0.04^a,A^	0.33 ± 0.09^a,A^	1.26 ± 0.47^a,A^	3.60 ± 0.31^a,A^
	7	0.18 ± 0.04^a,A^	0.20 ± 0.11^a,A^	0.35 ± 0.08^a,A^	0.96 ± 0.31^a,A^
	14	1.16 ± 0.21^a,B^	1.43 ± 0.15^a,B^	0.75 ± 0.06^a,A^	1.05 ± 0.08^a,A^
	21	0.49 ± 0.22^a,AB^	0.27 ± 0.07^a,A^	0.67 ± 0.39^a,A^	0.82 ± 0.08^a,A^
Hue	1	81.14 ± 0.50^a,A^	82.12 ± 0.89^ab,A^	84.37 ± 1.39^ab,A^	85.10 ± 2.07^b,A^
	7	82.27 ± 0.87^a,A^	82.43 ± 0.58^a,A^	84.45 ± 1.63^a,A^	85.03 ± 1.48^a,A^
	14	83.74 ± 0.42^a,A^	83.09 ± 1.63^a,A^	83.88 ± 0.83^a,A^	84.58 ± 2.69^a,A^
	21	81.03 ± 2.37^a,A^	81.38 ± 1.64^a,A^	85.22 ± 0.66^a,A^	85.39 ± 2.22^a,A^
*C*	1	33.06 ± 0.57^a,A^	33.95 ± 0.29^a,A^	34.83 ± 0.25^a,A^	33.66 ± 0.10^a,A^
	7	32.80 ± 1.40^ab,A^	33.45 ± 0.80^b,A^	32.10 ± 0.54^ab,A^	30.83 ± 1.26^a,A^
	14	34.04 ± 1.27^a,A^	33.09 ± 0.26^a,A^	33.47 ± 1.60^a,A^	33.05 ± 0.90^a,A^
	21	32.46 ± 0.38^a,A^	32.53 ± 0.30^a,A^	32.92 ± 1.29^a,A^	32.49 ± 0.55^a,A^

*Note*: Values indicated by different letters (a,b) in the same row are different from each other at *p* < 0.05 level. Values indicated by different letters (A,B) in the same column are different from each other at *p* < 0.05 level.

### 3.2 Total Phenolic Contents and Antioxidant Capacity

**FIGURE 2:**
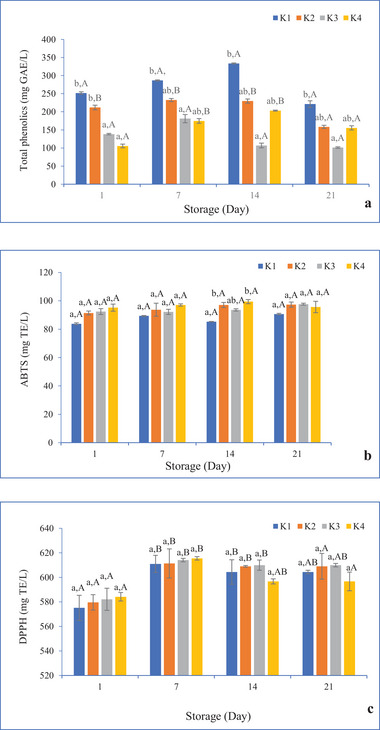
Total phenolic (a), ABTS (b), and DPPH (c) values of kefir samples containing 0% (K1), 5% (K2), 10% (K3), and 15% (K4) levels of Aloe vera. ABTS, 2,2′‐azino‐bis(3‐ethylbenzothiazoline‐6‐sulfonic acid); DPPH, 2,2‐diphenyl‐1‐picryhyldrazyl; GAE, gallic acid equivalent. Values indicated by different letters (a‐b) are different for the samples at P<0.05 level. Values indicated by different capitals (A‐B) are different for storage time at P<0.05 level.

The average total phenolic content of Aloe vera gel was 76.29 ± 1.14 mg GAE/L. Figure 2a shows that the kefir's total phenolic content ranged from 101.31 to 333.61 mg GAE/L. Aloe vera gel is known to have high levels of polyphenols (Nejatzadeh‐Barandozi 2013; El‐Sayed and El‐Sayed 2020), and adding Aloe vera gel to kefir boosted its total phenolic content (*p* < 0.05) on Days 1 and 21. The use of different formulations in kefir production significantly changed the total phenolic content of kefir (*p* < 0.001), whereas no statistical difference was found with the interaction of storage and *S* × *F* (*p* > 0.05). According to Yılmaz‐Ersan et al. (2018), the total phenolic content of kefir produced with sheep's and cow's milk was between 59.09 and 85.69 mg GAE/100 mL after 21 days of storage. In a study on the production of goat's milk kefir, the total phenolic content of the samples fluctuated between 59.66 and 69.96 mg GAE/100 mL during a 21‐day storage period (Yılmaz‐Ersan et al. 2016).

The average ABTS value of Aloe vera gel was 22.75 ± 1.10 mg TE/L. The ABTS values of kefir varied between 83.85 and 99.47 mg TE/L (Figure 2b). Because Aloe vera has an antioxidant effect, adding Aloe vera gel to kefir on the 14th day of storage raised its ABTS levels (*p* < 0.05) (Azari‐Anpar et al. 2017; Gao et al. 2019). No statistical difference was observed in the use of different formulas, storage, and the interaction *S* × *F* for the ABTS values of kefir (*p* > 0.05). Ozcan et al. (2019) found that the ABTS values for kefir from buffalo milk ranged between 11.12 and 13.69 mg TE/100 mL. The ABTS values of kefir made from sheep and cow milk ranged from 12.38 to 16.03 mg TE/100 mL, according to Yılmaz‐Ersan et al. (2018).

The average DPPH value of Aloe vera gel was 553.58 ± 2.09 mg TE/L. The DPPH values of kefir varied between 473.51 and 615.54 mg TE/L (Figure 2c). The DPPH values of samples K1 and K3 changed during storage (*p* < 0.05), and fluctuations were seen. The ability of the starter culture to maintain metabolic activity at low temperatures could be the cause of the increase in DPPH levels (and consequently antioxidant capacity). The decrease in DPPH levels could be due to the biodegradation of the antioxidant components during storage (Taşkın and Bağdatlıoğlu 2011). No statistical difference was observed with the use of different formulas and the interaction *S* × *F* for the DPPH values of kefir (*p* > 0.05), whereas storage was effective (*p* < 0.001). According to Ozcan et al. (2019), the DPPH values of kefir made from buffalo milk fluctuated between 1.73 and 4.49 mg TE/100 mL during the 21‐day storage period. In the study by Yılmaz‐Ersan et al. (2018), kefir made from sheep's and cow's milk had DPPH values between 2.79 and 6.55 mg TE/100 mL after 21 days of storage. The total phenolic content, ABTS, and DPPH values of kefir in the present study differed from the other studies summarized above. This could be due to differences in materials and production methods.

### 3.3 Volatile Compounds

**FIGURE 3:**
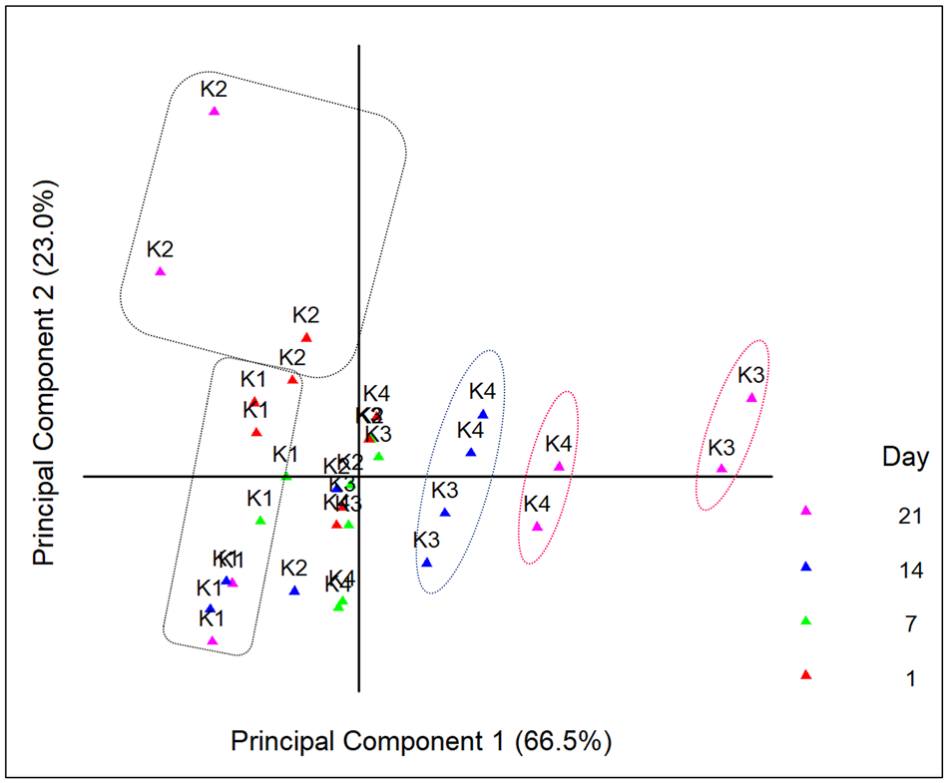
Principal component analysis (PCA) plot of volatile compounds of kefir samples containing 0% (K1), 5% (K2), 10% (K3), and 15% (K4) levels of Aloe vera.

A total of 36 and 44 compounds were determined in the samples of Aloe vera gel and kefir samples, respectively, including esters, acids, aldehydes, ketones, alcohols, terpenes, and miscellaneous compounds (presented in Tables S1 and S2). The differences in the volatile components of kefir were determined using PCA. The percentages for PC1 and PC2 were 66.5% and 23%, respectively (Figure 3). PCA showed that the volatile components of the different samples were found in particular regions. The different positions of the volatiles are due to the different concentrations of the volatiles in the samples. Furthermore, bornylene, ethyl acetate, and hexanal were the predominant compounds in the Aloe vera gel, whereas ethyl heptanoate, acetoin, and ethyl acetate were present in large quantities in kefir. The Aloe vera gel was found to have a positive effect on the content of volatile substances in the samples. Certain compounds were not found in the control sample (K1), but they were determined in the samples mixed with Aloe vera gel. These compounds included esters (methyl acetate, propyl acetate, ethyl butyrate, 3‐methyl‐1‐butyl acetate, and hexyl acetate), acids (2‐methylpentanoic acid), aldehydes (2‐methyl‐1‐butanal, 3‐methyl‐1‐butanal, octanal, and nonanal), and alcohols (isopropyl alcohol, 2‐butanol, isobutyl alcohol, 1‐hexanol, 2‐butanol, 2‐nonanol, and benzyl alcohol). Some of the volatiles (1‐hexanol, 3‐methyl‐1‐butanol, 3‐methyl‐1‐butanal, and nonanal) were present in high amounts in kefir samples and have already been identified in the literature (Duran et al. 2022; Rutkowska et al. 2022). Although 2‐heptanol, 2,3‐butanediol, and 1‐octanol were discovered in all samples, they were found in greater quantities in kefirs that had Aloe vera gel.

Sathyaprabha et al. (2010) reported that a total of 21 compounds were identified for Aloe vera, mainly hexadecanoic acid, oleic acid, and squalene. Similarly, 26 bioactive compounds were identified, with sitosterol, oleic acid, and octodecatrienoic acid methyl ester being the most important compounds for Aloe vera leaves (Arunkumar and Muthuselvam 2009). A comparison of the studies in the literature showed that the number of volatiles was higher for Aloe vera gel in the current study. This could be due to the different climatic zones and growing conditions of Aloe vera. According to Beirami‐Serizkani et al. (2021), kefir with Persian gum and microbial transglutaminase contains 51 volatiles, including nitrogen, CO_2_, alcohols, esters, acids, ketones, aldehydes, and terpenes. Aidarbekova and Aider (2022) reported that for kefir produced with electro‐activated whey, a total of 26 volatile aroma compounds were determined, including ketones, aldehydes, ethers, alcohols, acids, organic components, aromatic hydrocarbons, and CO_2_. The results of the present study demonstrated that the Aloe vera gel contributes to the volatile profile of kefir and were similar to other kefir studies in the literature.

### 3.4 Peptide Profiles

**FIGURE 4:**
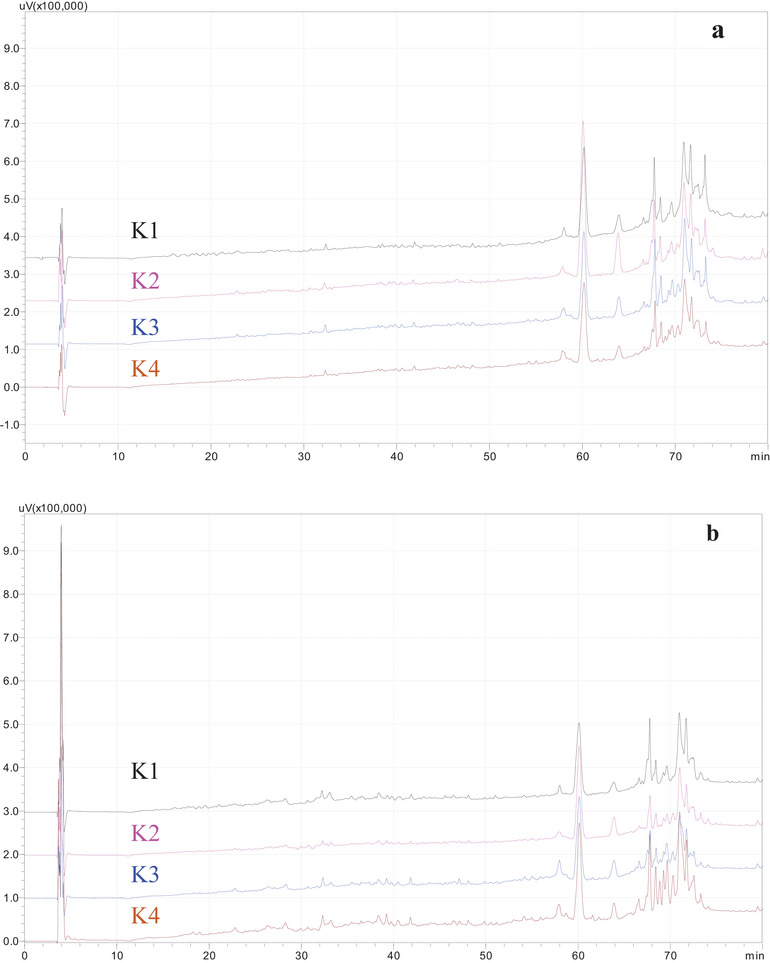
Peptide profiles of kefir samples containing 0% (K1), 5% (K2), 10% (K3), and 15% (K4) levels of Aloe vera after 1st (a) and 21st (b) days of storage.

The peptide profiles of kefir are shown in Figure 4 (for the 1st and 21st days of storage). The peak numbers and heights showed no significant differences between the individual kefir types, as the separate examination of the chromatograms revealed. Thus, it is concluded that Aloe vera gel did not influence the peptide profiles of kefir. The chromatograms’ co‐analysis revealed that the peaks’ height and number rose on the 21st day of storage. This is due to the formation of new peptides and an increase in peptide concentrations during storage. Ebner et al. (2015) determined the peptide profile of kefir using MALDI‐ToF‐MS. They reported that 230 different peptides were isolated by nano‐UPLC‐nano‐ESI‐MS/MS. In another study, the peptide profiles of kefir produced with supplemental cultures were analyzed on the 1st and 28th days, and there were only minor differences in the chromatograms and some storage‐related changes (Şanlı et al. 2018). The present study's conclusions are consistent with the other studies that were previously compiled.

### 3.5 Rheological Properties

**FIGURE 5:**
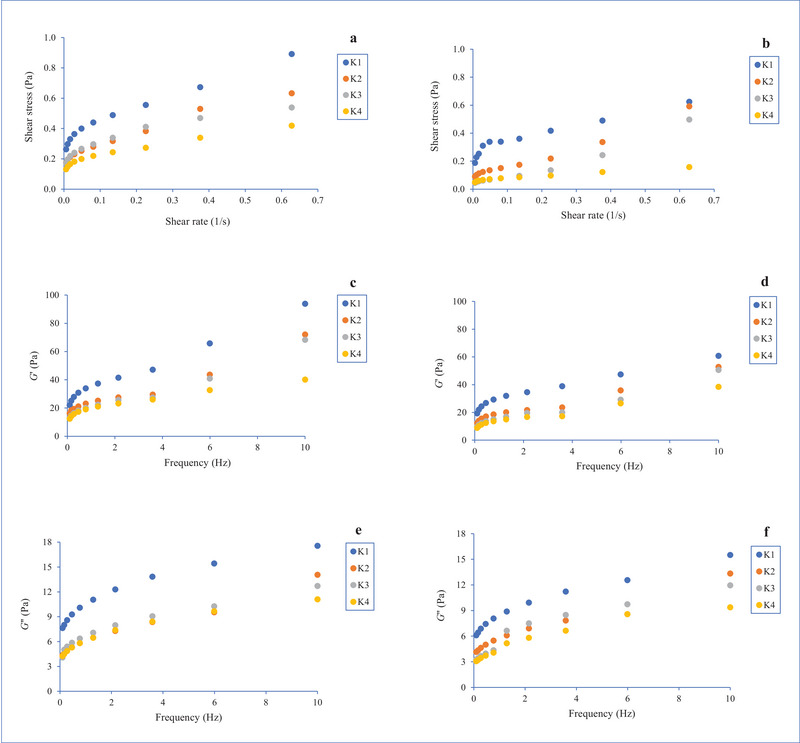
Shear stress and shear rate relationship of kefir samples containing 0% (K1), 5% (K2), 10% (K3), and 15% (K4) levels of Aloe vera after the 1st (a) and 21st (b) days of storage; *G*′ and frequency relationship of kefirs on the 1st (c) and 21st (d) days of storage; *G*″ and frequency relationship of kefirs on the 1st (e) and 21st (f) days of storage.

The influence of the addition of Aloe vera gel and storage on the flow properties of kefir was investigated. Figure 5a,b shows the evaluation of the correlation between the shear stress and the shear rate of kefir on Days 1 and 21. Kefir showed a non‐Newtonian flow behavior and pseudoplastic behavior. This is a typical flow property of fermented milk beverages and was found in previous studies such as Vimercati et al. (2020). After the addition of Aloe vera gel and storage, the shear stress values decreased. Aloe vera gel exhibited shear thinning (non‐Newtonian) behavior, as found by Lad and Murthy (2013). Aloe vera gel exhibits a weak and random polymer network due to the polysaccharide‐based gel network, and the gel structure deforms under shear stress (Choudhary et al. 2010). Therefore, the flow behavior of kefir can be negatively affected by Aloe vera gel, as found in the study by Azari‐Anpar et al. (2017).

The dynamic properties of kefir during storage were investigated. As a function of frequency, correlations between the elastic modulus (*G*′) and viscous modulus (*G*″) were found. The changes of the viscous and elastic modulus of kefir were measured on the 1st and 21st days of storage. Because the *G*′ values were greater than the *G*″ values and the *G*′/*G*″ ratio was less than 10, kefir had weak gelling characteristics unique to fermented milk beverages. The values of *G*′ and *G*″ of kefir increased with storage, as determined in Bensmira and Jiang's (2012) research. This increase is due to the metabolic activities of LAB (such as lowering pH and aggregation of casein micelles) during fermentation and storage (Bensmira et al. 2010). However, the *G*′ and *G*″ values of kefir dropped with the addition of Aloe vera gel. It was concluded that Aloe vera gel does not positively contribute to the rheological properties of kefir. This might be the result of mixing the Aloe vera gel before adding it to the inoculated milk and homogenizing the samples before fermentation to create a homogenous structure.

### 3.6 Microbiological Changes

The results of the microbiological analysis are shown in Table 3. The results met the requirements of the Turkish Food Codex for Fermented Dairy Products (Anonymous 2022) for the minimum concentrations of the total number of specific microorganisms and the total number of additional microorganisms and yeasts, which are 10^7^, 10^6^, and 10^4^ CFU/mL, respectively. The total number of aerobic mesophilic bacteria (TAMB) in kefir was between 3.27 and 5.83 log CFU/mL. The number of yeasts and molds in kefir was between 2.12 and 4.29 log CFU/mL. No statistical difference in yeast and mold counts was found when using different formulas, storage, and the interaction *S* × *F* (*p* > 0.05). The number of lactobacilli in kefir changed between 6.42 and 8.66 log CFU/mL. The number of lactococci in the kefirs ranged between 6.91 and 8.71 log CFU/mL. The kefir samples did not contain any coliform bacteria during storage. *E. coli* levels in the kefir varied between 0.00 and 3.21 log CFU/mL. The *E. coli* count in the K2‐encoded sample increased with the addition of Aloe vera gel (*p* < 0.05). The count for *E. coli* was higher in the samples spiked with Aloe vera gel at the beginning of storage. The presence of these microorganisms may be related to contamination during the extraction of Aloe vera gel (the leaves were only washed with water). The inhibition of *E. coli* during storage could be due to the antimicrobial effect of Aloe vera gel (Arunkumar and Muthuselvam 2009; Gao et al. 2019) and the production of hydrogen peroxide (H_2_O_2_) by LAB (Kıvanc and Yapıcı 2019). No statistical difference was observed with the use of different formulas and the interaction *S* × *F* for the number of TAMB, lactobacilli, lactococci, and *E. coli* (*p* > 0.05), whereas storage was effective (*p* < 0.05). The results of the microbiological analysis of kefir were similar to previous studies in the literature (Kıvanc and Yapıcı 2019; Turek and Wszolek 2022).

**TABLE 3:** Microbiological counts (log_10_ CFU/mL) of kefir samples containing 0% (K1), 5% (K2), 10% (K3), and 15% (K4) levels of Aloe vera.

Sample					
	Storage (day)	K1	K2	K3	K4
TAMB	1	4.27 ± 0.27^a,A^	4.18 ± 0.22^a,A^	4.01 ± 0.46^a,A^	3.91 ± 0.86^a,A^
	7	3.27 ± 0.82^a,A^	4.07 ± 0.21^a,A^	4.06 ± 0.89^a,A^	4.15 ± 0.84^a,A^
	14	4.57 ± 1.05^a,A^	4.40 ± 1.28^a,A^	4.64 ± 1.01^a,A^	4.78 ± 1.06^a,A^
	21	5.76 ± 0.89^a,A^	4.87 ± 0.32^a,A^	5.83 ± 0.71^a,A^	5.10 ± 0.42^a,A^
Yeasts and molds	1	3.10 ± 0.27^a,A^	3.69 ± 0.22^a,AB^	3.90 ± 0.50^a,A^	4.09 ± 0.32^a,A^
	7	3.98 ± 0.27^a,A^	4.26 ± 0.28^a,B^	4.12 ± 0.03^a,A^	4.29 ± 0.14^a,A^
	14	3.69 ± 0.30^a,A^	3.81 ± 0.16^a,AB^	3.85 ± 0.05^a,A^	3.77 ± 0.46^a,A^
	21	2.12 ± 0.01^a,A^	2.97 ± 0.80^a,A^	3.90 ± 0.10^a,A^	3.75 ± 0.24^a,A^
*Lactobacilli*	1	6.42 ± 0.18^a,A^	7.89 ± 1.02^a,AB^	7.50 ± 0.99^a,A^	7.31 ± 0.71^a,A^
	7	8.66 ± 0.29^a,A^	8.46 ± 0.13^a,B^	8.39 ± 0.06^a,A^	8.30 ± 0.03^a,A^
	14	7.82 ± 0.86^a,A^	7.71 ± 0.64^a,AB^	8.48 ± 0.49^a,A^	7.33 ± 0.97^a,A^
	21	7.13 ± 0.93^a,A^	6.53 ± 0.47^a,A^	7.54 ± 0.20^a,A^	7.07 ± 0.65^a,A^
*Lactococci*	1	8.64 ± 0.00^a,A^	8.43 ± 0.05^a,A^	8.44 ± 0.00^a,A^	6.91 ± 0.63^a,A^
	7	8.71 ± 0.19^b,A^	8.55 ± 0.11^ab,A^	8.46 ± 0.07^ab,A^	8.35 ± 0.07^a,B^
	14	8.22 ± 0.76^a,A^	7.97 ± 0.70^a,A^	7.90 ± 0.22^a,A^	7.98 ± 0.77^a,AB^
	21	7.25 ± 0.29^a,A^	6.97 ± 0.07^a,A^	7.50 ± 0.16^a,A^	7.22 ± 0.17^a,AB^
Total coliforms	1	ND	ND	ND	ND
	7	ND	ND	ND	ND
	14	ND	ND	ND	ND
	21	ND	ND	ND	ND
*Escherichia coli*	1	1.43 ± 0.55^a,B^	3.21 ± 0.52^c,B^	3.17 ± 0.22^c,B^	2.94 ± 0.50^b,B^
	7	ND	ND	ND	ND
	14	ND	ND	ND	ND
	21	ND	ND	ND	ND

*Note*: Values indicated by different letters (a–c) in the same row are different from each other at *p* < 0.05 level. Values indicated by different letters (A,B) in the same column are different from each other at *p* < 0.05 level.

Abbreviation: ND, not detected.

### 3.7 Sensory Properties

**FIGURE 6:**
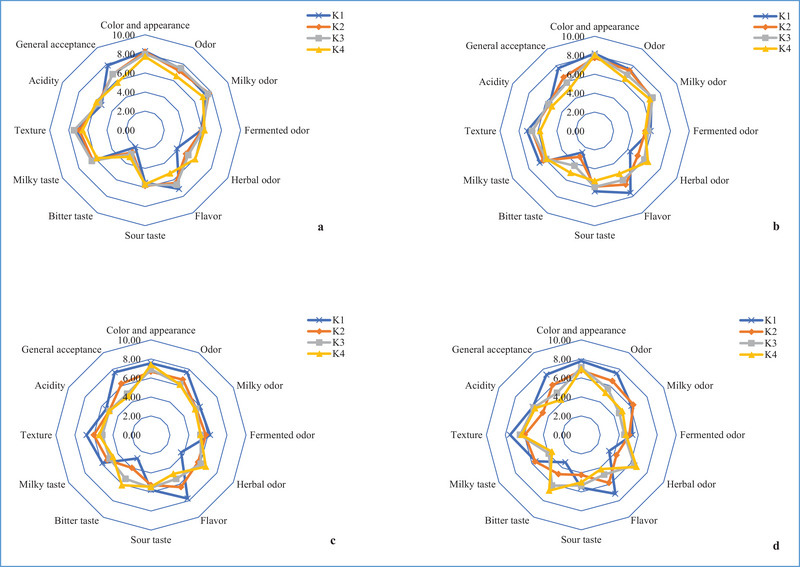
Spider plot for flavor attributes of kefir samples containing 0% (K1), 5% (K2), 10% (K3), and 15% (K4) levels of Aloe vera after the 1st (a), 7th (b), 14th (c), and 21st (d) days of storage.

The spider plots for the taste characteristics of kefir are shown in Figure 6. Panelists rated the color and appearance of kefir as 6.67–8.28, and storage was statistically effective (*p* < 0.05). Evaluations of the odor characteristics ranged from 5.11 to 7.61, and the addition of Aloe vera gel and storage had a significant impact (*p* < 0.001). The milky odor of kefir was rated between 4.61 and 7.72, and storage significantly changed the milky odor scores (*p* < 0.001). The fermented odor characteristic of kefir was evaluated as 4.56–6.22, and no statistical difference was found (*p* > 0.05). The characteristic herbal odor of kefir was evaluated as 3.39–6.72, and using different formulations considerably changed the odor scores (*p* < 0.01). The taste characteristics of the kefir were assessed with values between 4.14 and 7.72, and using different formulas significantly influenced the taste values (*p* < 0.001). The sour taste of kefir was rated with values between 4.22 and 6.38, and no statistical difference was seen (*p* > 0.05). The scores for the bitter taste of kefir ranged from 2.06 to 6.78 and using different formulations and storage significantly impacted the bitter taste values (*p* < 0.001). The milky taste of kefir was rated at 3.56–6.71, and the use of different formulations (*p* < 0.05) and storage (*p* < 0.01) considerably changed the ratings of the lactic taste. The texture characteristics of the kefir samples were rated 5.44–7.50, and the use of different formulations and storage significantly changed the texture values (*p* < 0.05). The acidity of kefir ranged from 4.67 to 5.89, and no statistical difference was found (*p* > 0.05). The general acceptability characteristic of kefir ranged from 4.28 to 7.83, and the use of different formulations (*p* < 0.001) and storage (*p* < 0.05) significantly changed the general acceptability. It was found that the scores of sensory characteristics of kefir samples dropped as more Aloe vera gel was applied. This might be due to the limited use of Aloe vera gel in foods. Aloe vera gel added to kefir samples may appear different to the panelists in terms of sensory properties; therefore, the control sample received higher scores. On the other hand, it was also observed that Aloe vera gel–applied samples scored well. The findings align with previous research on kefir in the literature, such as Vimercati et al. (2020) and Beirami‐Serizkani et al. (2021).

## 4 Conclusions

In this study, a novel kefir enriched with Aloe vera gel was produced. The physical, chemical, microbiological, rheological, and sensory properties were evaluated during storage. The analytical results showed that the kefir sample (code K3) enriched with 10% Aloe vera gel was excellent in terms of composition, antioxidant activity, volatile compound profile, and sensory properties. The use of Aloe vera gel is promising to increase kefir consumption and promote consumer purchase intention for plant‐based dairy products. In conclusion, it is recommended to industrially produce this innovative fermented milk product to utilize the health benefits of kefir and Aloe vera. A further study should be conducted to determine the antimicrobial activity and shelf life of the product. Furthermore, the usage of Aloe vera gel powder in the production may improve the bioactive and sensory properties, flow characteristics, and texture of kefir.

## Author Contributions

**İsra Yiğitvar**: conceptualization, methodology, data curation, software, investigation, formal analysis, writing–original draft. **Ali Adnan Hayaloğlu**: conceptualization, writing–review and editing, project administration, supervision, validation, investigation, funding acquisition, visualization.

## Ethics Statement

This research closely followed the ethical standards set by Inönü University's Institutional Review Board (IRB) in Malatya, Türkiye. The university granted formal approval before the research could begin, with reference number #2082. Every procedure that involved human subjects was carefully carried out in compliance with the university's ethical guidelines. The university approved the study protocol after it was thoroughly reviewed. Each author attests that there are no human or animal trial experiments in the study described in the manuscript.

## Consent

Every participant gave their informed consent before beginning the study, guaranteeing their voluntary participation and comprehension of the goals of the investigation. All participants in the sensory evaluation signed a written informed consent form to the university‐approved protocol, which adhered to the ethical guidelines established in the Declaration of Helsinki and its subsequent amendments or similar standards.

## Conflicts of Interest

The authors declare no conflicts of interest.

## Supporting information



Supporting Information
