# Supplementary material for: Incorporation of Aloe Vera Gel Into Kefir: Impact on Volatile Composition, Rheological, Techno‐Functional, and Microbiological Properties
Source: J Food Sci. 2025 May 28;90(5):e70292. doi: 10.1111/1750-3841.70292 (PMC12117997; doi:10.1111/1750-3841.70292)
Supplement: Supplementary file 1 — Supporting Information [file JFDS-90-0-s001.docx]

**Supplementary Table 1.** Volatile compounds in Aloe vera gel (µg/g)

| Compounds | RI | Concentration± SD |
| --- | --- | --- |
| Methyl acetate | 864 | 0.11±0.04 |
| Ethyl acetate | 900 | 9.63±2.52 |
| 3-Methylbutanal | 910 | 1.58±0.45 |
| Pentanal | 922 | 0.50±0.43 |
| Ethanol | 937 | 0.79±0.13 |
| α-Pinene | 1021 | 0.25±0.04 |
| Toluene | 1039 | 1.77±0.24 |
| Hexanal | 1080 | 2.93±0.84 |
| β-Pinene | 1102 | 0.24±0.19 |
| β-Myrcene | 1149 | 0.33±0.06 |
| 3-Methyl-1-butanol | 1208 | 2.82±0.63 |
| Eucalyptol | 1212 | 0.32±0.24 |
| 2-Hexenal | 1225 | 0.85±0.48 |
| 1-Pentanol | 1251 | 0.22±0.07 |
| Styrene | 1258 | 0.43±0.14 |
| Acetoin | 1289 | 0.20±0.12 |
| 6-Methyl-5-hepten-2-one | 1343 | 0.10±0.02 |
| Ethyl trichloroacetate | 1372 | 0.46±0.17 |
| Isobutyric acid | 1393 | 0.23±0.10 |
| Nonanal | 1399 | 0.23±0.16 |
| Ethyl heptanonate | 1411 | 0.45±0.11 |
| Decanal | 1428 | 0.14±0.01 |
| Tetradecanoic acid | 1430 | 0.46±0.43 |
| Tetrahydrolinalool | 1431 | 0.18±0.13 |
| 1-Undecanol | 1439 | 0.11±0.03 |
| Dihydromyrcenol | 1443 | 0.10±0.02 |
| Methyl trichloroacetate | 1455 | 2.14±1.91 |
| Suppl. Table 1 (Continued)  Compounds | RI | Concentration ± SD |
| Sabinene | 1465 | 0.05±0.01 |
| Bornylene | 1490 | 9.65±0.85 |
| 2-Ethyl-1-hexanol | 1492 | 0.44±0.25 |
| Benzene-1,2-dicarboxylic acid | 1510 | 0.33±0.19 |
| Benzaldehyde | 1543 | 0.38±0.19 |
| Linalool | 1549 | 0.57±0.38 |
| Benzeneacetic acid | 1552 | 0.50±0.36 |
| α-Terpineol | 1712 | 0.20±0.11 |
| 2-Propenoic acid | 2104 | 0.38±0.07 |

RI: Retention Index

**Supplementary Table 2.** Volatile compounds (µg/g) in kefir samples containing 0% (K1), 5% (K2), 10% (K3) and 15% (K4) levels of Aloe vera

| Compounds | RI | Storage (day) | Sample | | | |
| --- | --- | --- | --- | --- | --- | --- |
|  |  |  | K1 | K2 | K3 | K4 |
| Acetone | 795 | 1 | 16.37±0.36^c,C^ | *ND* | 10.08±0.03^b,AB^ | 9.83±0.14^b,B^ |
|  |  | 7 | 21.35±0.29^b,D^ | 5.80±0.40^a,B^ | 51.60±3.40^c,C^ | 11.48±0.16^a,C^ |
|  |  | 14 | *ND* | 13.87±0.40^b,C^ | 13.62±0.65^b,B^ | 13.24±0.24^b,D^ |
|  |  | 21 | 14.18±0.08^b,B^ | 26.87±0.89^c,D^ | 4.38±0.26^a,A^ | 5.23±0.22^a,A^ |
| Methyl acetate | 864 | 1 | *ND* | 0.76±0.04^a,B^ | 1.11±0.09^ab,B^ | 2.71±0.67^b,A^ |
|  |  | 7 | *ND* | 0.71±0.01^a,B^ | 1.60±0.23^b,B^ | 1.61±0.34^b,A^ |
|  |  | 14 | *ND* | 1.13±0.05^b,C^ | 3.13±0.13^c,C^ | 3.09±0.08^c,A^ |
|  |  | 21 | *ND* | *ND* | *ND* | 2.29±0.28^b,A^ |
| Ethyl acetate | 900 | 1 | 66.77±1.56^c,B^ | *ND* | 88.67±1.13^d,B^ | 48.55±2.23^b,A^ |
|  |  | 7 | 122.72±2.01^b,C^ | 71.57±2.25^a,D^ | 127.05±2.18^b,C^ | 78.74±2.33^a,B^ |
|  |  | 14 | 57.66±2.19^b,A^ | 45.01±0.91^a,C^ | 85.21±2.20^c,B^ | 76.60±5.13^c,B^ |
|  |  | 21 | 61.94±2.26^b,AB^ | 14.32±0.63^a,B^ | 73.08±3.08^c,A^ | 67.40±3.82^bc,B^ |
| 2-Methyl-1-butanal | 906 | 1 | *ND* | *ND* | *ND* | *ND* |
|  |  | 7 | *ND* | *ND* | *ND* | *ND* |
|  |  | 14 | *ND* | *ND* | 1.14±0.11^b,B^ | *ND* |
|  |  | 21 | *ND* | *ND* | 3.34±0.11^c,C^ | 0.92±0.01^b,B^ |
| 3-Methyl-1-butanal | 910 | 1 | *ND* | *ND* | *ND* | *ND* |
|  |  | 7 | *ND* | *ND* | *ND* | *ND* |
|  |  | 14 | *ND* | 0.70±0.02^b,B^ | 1.89±0.68^c,B^ | 0.86±0.11^b,B^ |
|  |  | 21 | *ND* | *ND* | 3.71±0.09^c,C^ | 1.11±0.20^b,C^ |
| Ethanol | 937 | 1 | 8.97±1.04^b,AB^ | 9.37±0.19^b,BC^ | 4.66±0.06^a,B^ | 5.33±0.19^a,A^ |
|  |  | 7 | 13.63±0.60^c,B^ | 2.64±0.63^a,A^ | 18.10±0.42^d,D^ | 8.19±0.19^b,B^ |
|  |  | 14 | 13.75±2.30^b,B^ | 7.56±0.69^a,B^ | 12.45±0.45^ab,C^ | 10.47±0.55^ab,C^ |
|  |  | 21 | 7.09±0.51^b,A^ | 11.14±0.14^c,C^ | *ND* | 12.01±0.03^c,D^ |

Suppl. Table 2 (Continued)

| Compounds | RI | Storage (day) | Sample | | | |
| --- | --- | --- | --- | --- | --- | --- |
|  |  |  | K1 | K2 | K3 | K4 |
| 2,3-Butanedione | 958 | 1 | 16.58±0.59^b,C^ | 13.48±0.22^b,B^ | 7.67±1.17^a,B^ | 8.35±0.85^a,B^ |
|  |  | 7 | 4.72±0.26^c,A^ | 1.81±0.01^b,A^ | *ND* | 1.10±0.10^b,A^ |
|  |  | 14 | 8.15±0.67^c,B^ | 3.64±0.12^b,A^ | *ND* | *ND* |
|  |  | 21 | 4.78±0.21^b,A^ | 31.32±1.56^c,C^ | *ND* | *ND* |
| Methyl butyrate | 968 | 1 | *ND* | *ND* | 0.51±0.04^b,B^ | 0.91±0.06^c,B^ |
|  |  | 7 | *ND* | 0.56±0.01^b,B^ | 1.98±0.22^c,C^ | 0.69±0.11^b,B^ |
|  |  | 14 | 0.99±0.01^c,C^ | 0.57±0.02^b,B^ | *ND* | *ND* |
|  |  | 21 | 0.56±0.05^b,B^ | *ND* | *ND* | *ND* |
| Propyl acetate | 971 | 1 | *ND* | *ND* | *ND* | *ND* |
|  |  | 7 | *ND* | *ND* | *ND* | *ND* |
|  |  | 14 | *ND* | *ND* | 1.15±0.05^c,B^ | 0.32±0.01^b,B^ |
|  |  | 21 | *ND* | *ND* | 2.07±0.14^c,C^ | 0.69±0.03^b,C^ |
| 2-Pentanone | 974 | 1 | *ND* | *ND* | *ND* | *ND* |
|  |  | 7 | 3.85±0.13^c,B^ | 1.34±0.38^b,AB^ | *ND* | 1.24±0.21^b,B^ |
|  |  | 14 | 4.78±0.78^b,B^ | 2.92±0.91^ab,B^ | 1.71±0.19^a,B^ | 0.92±0.02^a,B^ |
|  |  | 21 | 3.85±0.15^c,B^ | 6.81±0.10^d,C^ | *ND* | 0.96±0.03^b,B^ |
| 2-Butanol | 1021 | 1 | *ND* | *ND* | *ND* | *ND* |
|  |  | 7 | *ND* | *ND* | *ND* | 0.53±0.05^b,B^ |
|  |  | 14 | *ND* | *ND* | 5.39±0.62^b,B^ | 2.86±0.01^bc,C^ |
|  |  | 21 | *ND* | *ND* | 31.42±1.21^c,C^ | 5.44±0.06^c,D^ |
| Ethyl butyrate | 1024 | 1 | *ND* | *ND* | *ND* | *ND* |
|  |  | 7 | *ND* | *ND* | 3.21±0.36^b,B^ | 0.22±0.01^a,B^ |
|  |  | 14 | *ND* | *ND* | *ND* | 0.25±0.01^b,B^ |
|  |  | 21 | *ND* | *ND* | *ND* | 0.38±0.03^b,C^ |

Suppl. Table 2 (Continued)

| Compounds | RI | Storage (day) | Sample | | | |
| --- | --- | --- | --- | --- | --- | --- |
|  |  |  | K1 | K2 | K3 | K4 |
| Hexanal | 1080 | 1 | *ND* | *ND* | 2.40±0.36^b,A^ | 4.29±0.28^c,B^ |
|  |  | 7 | 0.87±0.01^a,B^ | 1.08±0.07^a,B^ | 3.72±0.27^b,B^ | 1.09±0.10^a,A^ |
|  |  | 14 | 0.99±0.01^a,B^ | 2.04±0.08^b,C^ | 5.45±0.43^c,C^ | 7.08±0.16^d,C^ |
|  |  | 21 | 0.99±0.06^a,B^ | 4.14±0.11^b,D^ | 8.83±0.24^c,D^ | 8.68±0.13^c,D^ |
| Isobutyl alcohol | 1089 | 1 | *ND* | *ND* | *ND* | *ND* |
|  |  | 7 | *ND* | *ND* | *ND* | *ND* |
|  |  | 14 | *ND* | *ND* | *ND* | *ND* |
|  |  | 21 | *ND* | *ND* | 3.65±0.20^b,B^ | *ND* |
| β-Pinene | 1102 | 1 | *ND* | *ND* | *ND* | *ND* |
|  |  | 7 | *ND* | *ND* | *ND* | *ND* |
|  |  | 14 | *ND* | *ND* | *ND* | 1.09±0.12^c,B^ |
|  |  | 21 | 0.52±0.01^b,B^ | *ND* | *ND* | 1.84±0.11^d,C^ |
| 2-Pentanol | 1119 | 1 | *ND* | *ND* | *ND* | *ND* |
|  |  | 7 | *ND* | *ND* | *ND* | *ND* |
|  |  | 14 | *ND* | 1.66±0.04^b,B^ | *ND* | *ND* |
|  |  | 21 | *ND* | *ND* | 2.38±1.07^b,B^ | 0.42±0.02^ab,B^ |
| 3-Methyl-1-butanol acetate | 1125 | 1 | *ND* | *ND* | *ND* | *ND* |
|  |  | 7 | *ND* | *ND* | *ND* | *ND* |
|  |  | 14 | *ND* | *ND* | 1.94±0.21^b,B^ | 0.41±0.03^a,B^ |
|  |  | 21 | *ND* | *ND* | 11.00±0.12^c,C^ | 2.23±0.21^b,C^ |
| β-Mirsen | 1149 | 1 | *ND* | *ND* | *ND* | *ND* |
|  |  | 7 | *ND* | *ND* | *ND* | *ND* |
|  |  | 14 | *ND* | *ND* | *ND* | *ND* |
|  |  | 21 | 0.79±0.03^b,B^ | *ND* | *ND* | *ND* |

Suppl. Table 2 (Continued)

| Compounds | RI | Storage (day) | Sample | | | |
| --- | --- | --- | --- | --- | --- | --- |
|  |  |  | K1 | K2 | K3 | K4 |
| 2-Heptanone | 1186 | 1 | 19.33±0.37^c,C^ | 14.71±0.71^b,B^ | *ND* | *ND* |
|  |  | 7 | 13.40±0.60^c,A^ | 5.73±0.28^b,A^ | *ND* | *ND* |
|  |  | 14 | 22.39±0.43^c,D^ | 7.73±0.23^b,A^ | *ND* | *ND* |
|  |  | 21 | 15.91±0.11^b,B^ | 30.00±0.66^c,C^ | *ND* | *ND* |
| 3-Methyl-1-butanol | 1208 | 1 | *ND* | *ND* | *ND* | *ND* |
|  |  | 7 | *ND* | *ND* | 0.97±0.03^c,A^ | 0.61±0.07^b,A^ |
|  |  | 14 | *ND* | *ND* | 18.25±2.22^c,B^ | 8.39±1.25^b,B^ |
|  |  | 21 | *ND* | 3.29±0.29^a,B^ | 153.34±5.98^c,C^ | 29.20±0.80^b,C^ |
| Eucalyptol | 1212 | 1 | *ND* | *ND* | *ND* | *ND* |
|  |  | 7 | *ND* | *ND* | *ND* | *ND* |
|  |  | 14 | *ND* | *ND* | 0.59±0.02^b,B^ | 0.75±0.05^c,B^ |
|  |  | 21 | *ND* | *ND* | *ND* | *ND* |
| Ethyl hexanoate | 1235 | 1 | 1.15±0.01^a,A^ | 2.18±0.12^b,B^ | 1.40±0.23^a,A^ | 1.25±0.13^a,A^ |
|  |  | 7 | 1.09±0.11^a,A^ | 1.29±0.04^a,A^ | 1.15±0.23^a,A^ | 1.09±0.06^a,A^ |
|  |  | 14 | 1.06±0.10^a,A^ | 1.08±0.04^ab,A^ | 1.39±0.07^b,A^ | 1.23±0.10^ab,A^ |
|  |  | 21 | 1.08±0.01^a,A^ | 1.32±0.24^a,A^ | 1.60±0.32^a,A^ | 1.44±0.33^a,A^ |
| 1-Pentanol | 1251 | 1 | *ND* | 3.57±0.43^b,B^ | *ND* | *ND* |
|  |  | 7 | *ND* | *ND* | *ND* | *ND* |
|  |  | 14 | 0.78±0.03^b,B^ | *ND* | 0.80±0.04^b,B^ | 1.31±0.06^c,B^ |
|  |  | 21 | *ND* | *ND* | *ND* | *ND* |
| Hexyl acetate | 1276 | 1 | *ND* | *ND* | *ND* | *ND* |
|  |  | 7 | *ND* | *ND* | *ND* | *ND* |
|  |  | 14 | *ND* | *ND* | 0.85±0.04^b,B^ | *ND* |
|  |  | 21 | *ND* | *ND* | 6.08±0.07^c,C^ | 2.00±0.13^b,B^ |

Suppl. Table 2 (Continued)

| Compounds | RI | Storage (day) | Sample | | | |
| --- | --- | --- | --- | --- | --- | --- |
|  |  |  | K1 | K2 | K3 | K4 |
| Acetoin | 1289 | 1 | 169.28±8.03^c,B^ | 127.49±3.13^b,C^ | 46.87±1.46^a,C^ | 39.22±0.20^a,C^ |
|  |  | 7 | 124.89±4.24^c,A^ | 42.92±1.29^b,A^ | 35.53±1.32^b,B^ | 9.62±0.39^a,B^ |
|  |  | 14 | 131.93±1.39^c,A^ | 67.58±0.63^b,B^ | *ND* | *ND* |
|  |  | 21 | 123.90±1.32^b,A^ | 326.33±4.69^c,D^ | *ND* | *ND* |
| Octanal | 1294 | 1 | *ND* | *ND* | 0.32±0.01^b,B^ | 0.60±0.02^c,B^ |
|  |  | 7 | *ND* | *ND* | *ND* | *ND* |
|  |  | 14 | *ND* | *ND* | 0.57±0.01^b,C^ | 0.68±0.04^c,B^ |
|  |  | 21 | *ND* | *ND* | 1.74±0.11^b,D^ | 2.82±0.07^c,C^ |
| 2-Heptanol | 1320 | 1 | *ND* | *ND* | 2.09±0.05^c,A^ | 1.49±0.01^b,A^ |
|  |  | 7 | 1.14±0.14^a,B^ | *ND* | 6.50±2.39^b,A^ | 3.62±0.11^ab,B^ |
|  |  | 14 | 1.35±0.05^a,B^ | 2.32±0.22^a,B^ | 13.24±0.24^c,B^ | 6.59±0.41^b,C^ |
|  |  | 21 | 1.14±0.11^a,B^ | 5.97±0.04^b,C^ | 18.15±0.85^d,B^ | 9.08±0.02^c,D^ |
| 1-Hexanol | 1359 | 1 | *ND* | *ND* | *ND* | *ND* |
|  |  | 7 | *ND* | *ND* | *ND* | *ND* |
|  |  | 14 | *ND* | 17.58±0.18^b,B^ | 70.98±0.40^c,B^ | 74.42±0.07^c,B^ |
|  |  | 21 | *ND* | *ND* | 316.87±3.27^c,C^ | 171.24±0.68^b,C^ |
| 2-Nonanone | 1396 | 1 | 6.70±0.28^c,C^ | 4.78±0.17^b,B^ | 1.76±0.03^a,B^ | 1.47±0.12^a,C^ |
|  |  | 7 | 3.06±0.08^c,A^ | 1.50±0.03^b,A^ | 2.96±0.14^c,C^ | 0.96±0.01^a,B^ |
|  |  | 14 | 4.12±0.16^d,AB^ | 2.65±0.03^c,A^ | 1.67±0.16^b,B^ | *ND* |
|  |  | 21 | 5.09±0.55^b,B^ | 10.63±0.67^c,C^ | *ND* | *ND* |
| Nonanal | 1399 | 1 | *ND* | 1.08±0.08^b,C^ | 1.05±0.04^b,C^ | 1.24±0.30^b,AB^ |
|  |  | 7 | *ND* | 0.70±0.02^b,B^ | 0.86±0.05^b,B^ | 1.29±0.10^c,AB^ |
|  |  | 14 | *ND* | *ND* | *ND* | 0.87±0.03^b,A^ |
|  |  | 21 | *ND* | *ND* | *ND* | 1.50±0.22^b,B^ |

Suppl. Table 2 (Continued)

| Compounds | RI | Storage (day) | Sample | | | |
| --- | --- | --- | --- | --- | --- | --- |
|  |  |  | K1 | K2 | K3 | K4 |
| Methyl 2-methylpentanoate | 1400 | 1 | *ND* | 0.88±0.01^b,A^ | 0.88±0.02^b,A^ | 0.88±0.03^b,A^ |
|  |  | 7 | 1.07±0.07^a,B^ | 0.93±0.09^a,A^ | 0.91±0.04^a,A^ | 1.01±0.14^a,A^ |
|  |  | 14 | 0.96±0.04^a,B^ | 1.16±0.04^b,A^ | 1.20±0.02^b,B^ | 0.99±0.01^a,A^ |
|  |  | 21 | 1.10±0.32^a,B^ | 1.17±0.13^a,A^ | 1.26±0.04^a,B^ | 1.20±0.30^a,A^ |
| Octanoic acid, methyl ester | 1417 | 1 | *ND* | *ND* | *ND* | *ND* |
|  |  | 7 | *ND* | *ND* | *ND* | *ND* |
|  |  | 14 | *ND* | *ND* | *ND* | *ND* |
|  |  | 21 | *ND* | *ND* | 1.41±0.23^c,B^ | 0.85±0.04^b,B^ |
| 4-Methyl-1-hexanol | 1421 | 1 | *ND* | *ND* | *ND* | *ND* |
|  |  | 7 | *ND* | *ND* | *ND* | *ND* |
|  |  | 14 | *ND* | *ND* | *ND* | *ND* |
|  |  | 21 | *ND* | *ND* | 1.83±0.03^b,B^ | *ND* |
| Butyl propyl ketone | 1431 | 1 | *ND* | *ND* | 0.38±0.03^c,B^ | 0.28±0.01^b,B^ |
|  |  | 7 | *ND* | 0.37±0.04^b,B^ | *ND* | *ND* |
|  |  | 14 | *ND* | *ND* | *ND* | *ND* |
|  |  | 21 | *ND* | *ND* | *ND* | *ND* |
| Tetrahydrolinalool | 1435 | 1 | *ND* | *ND* | *ND* | *ND* |
|  |  | 7 | *ND* | *ND* | *ND* | *ND* |
|  |  | 14 | *ND* | *ND* | *ND* | *ND* |
|  |  | 21 | *ND* | *ND* | *ND* | 12.75±0.75^b,B^ |
| Acetic acid | 1457 | 1 | 59.57±1.43^a,A^ | 121.99±8.66^b,C^ | 45.15±1.17^a,A^ | 47.73±2.35^a,B^ |
|  |  | 7 | 71.53±0.95^d,B^ | 49.86±0.32^b,A^ | 57.72±0.31^c,B^ | 25.35±0.35^a,A^ |
|  |  | 14 | 74.59±0.09^c,B^ | 58.06±0.25^a,AB^ | 87.54±0.04^d,C^ | 69.71±0.08^b,C^ |
|  |  | 21 | 61.16±0.14^c,A^ | 73.12±2.87^d,B^ | 44.58±1.63^b,A^ | 25.13±2.41^a,A^ |

Suppl. Table 2 (Continued)

| Compounds | RI | Storage (day) | Sample | | | |
| --- | --- | --- | --- | --- | --- | --- |
|  |  |  | K1 | K2 | K3 | K4 |
| Bornylene | 1490 | 1 | *ND* | 2.69±0.38^ab,B^ | 3.55±0.06^b,B^ | 4.09±0.09^bc,B^ |
|  |  | 7 | 3.99±0.58^b,C^ | *ND* | 14.87±0.91^d,D^ | 4.48±0.44^bc,B^ |
|  |  | 14 | 1.49±0.14^a,B^ | 2.09±0.08^ab,B^ | *ND* | 4.07±0.33^bc,B^ |
|  |  | 21 | *ND* | *ND* | 6.90±0.39^c,C^ | *ND* |
| 2-Ethyl-1-hexanol | 1492 | 1 | 1.69±0.19^c,D^ | *ND* | 1.08±0.04^b,C^ | 1.06±0.00^b,A^ |
|  |  | 7 | 1.15±0.03^a,C^ | 0.92±0.02^a,C^ | 2.76±0.16^b,D^ | 0.83±0.02^a,A^ |
|  |  | 14 | 0.77±0.01^b,B^ | 0.60±0.05^a,B^ | 0.67±0.02^ab,B^ | 0.96±0.01^c,A^ |
|  |  | 21 | *ND* | *ND* | *ND* | 3.25±1.48^b,B^ |
| 2-Nonanol | 1520 | 1 | *ND* | *ND* | *ND* | *ND* |
|  |  | 7 | *ND* | *ND* | *ND* | 0.46±0.02^b,B^ |
|  |  | 14 | *ND* | *ND* | 1.94±0.02^b,B^ | 1.81±0.03^b,C^ |
|  |  | 21 | *ND* | *ND* | 5.05±0.05^c,C^ | 2.75±0.03^b,D^ |
| 2,3-Butanediol | 1521 | 1 | *ND* | *ND* | 5.30±1.13^b,B^ | 0.85±0.03^a,A^ |
|  |  | 7 | *ND* | 1.27±0.11^b,C^ | 3.02±0.22^c,A^ | 3.28±0.59^c,B^ |
|  |  | 14 | 0.66±0.05^a,B^ | 0.93±0.03^a,B^ | 4.48±0.36^b,A^ | 4.73±0.20^b,C^ |
|  |  | 21 | *ND* | *ND* | 6.27±1.14^c,B^ | 3.16±0.18^b,B^ |
| Benzaldehyde | 1543 | 1 | *ND* | 1.35±0.08^a,AB^ | 1.50±0.25^a,A^ | 5.88±0.74^b,B^ |
|  |  | 7 | 1.35±0.14^a,B^ | 0.72±0.01^a,A^ | 4.15±0.65^b,A^ | 1.71±0.22^a,A^ |
|  |  | 14 | *ND* | 2.76±0.44^b,B^ | 10.08±0.43^c,B^ | 19.35±0.84^d,C^ |
|  |  | 21 | 2.50±0.14^a,C^ | 17.55±0.81^b,C^ | 25.89±1.38^c,C^ | 39.18±0.44^d,D^ |
| Linalool | 1549 | 1 | *ND* | 0.54±0.04^b,B^ | *ND* | *ND* |
|  |  | 7 | *ND* | *ND* | *ND* | *ND* |
|  |  | 14 | *ND* | *ND* | *ND* | *ND* |
|  |  | 21 | *ND* | 1.49±0.13^c,C^ | 1.84±0.05^d,B^ | *ND* |

Suppl. Table 2 (Continued)

| Compounds | RI | Storage (Day) | Sample | | | |
| --- | --- | --- | --- | --- | --- | --- |
|  |  |  | K1 | K2 | K3 | K4 |
| Benzeneacetic acid | 1552 | 1 | *ND* | *ND* | *ND* | *ND* |
|  |  | 7 | *ND* | *ND* | *ND* | *ND* |
|  |  | 14 | *ND* | *ND* | 1.00±0.08^b,B^ | *ND* |
|  |  | 21 | 0.97±0.02^b,B^ | 1.82±0.11^c,B^ | *ND* | *ND* |
| 1-Octanol | 1562 | 1 | *ND* | *ND* | *ND* | 0.55±0.05^b,A^ |
|  |  | 7 | *ND* | *ND* | 1.04±0.04^b,B^ | 11.48±0.04^c,D^ |
|  |  | 14 | *ND* | 0.59±0.08^b,B^ | 1.70±0.05^c,C^ | 1.82±0.01^c,B^ |
|  |  | 21 | 0.30±0.02^a,B^ | 2.37±0.01^b,C^ | 14.65±0.05^d,D^ | 8.15±0.05^c,C^ |
| 2-Undecanone | 1608 | 1 | 1.34±0.16^c,C^ | 0.92±0.03^b,C^ | 0.46±0.01^a,B^ | 0.46±0.02^a,B^ |
|  |  | 7 | 0.51±0.04^b,A^ | *ND* | *ND* | *ND* |
|  |  | 14 | 0.61±0.06^c,AB^ | 0.36±0.03^b,B^ | *ND* | 0.41±0.07^b,B^ |
|  |  | 21 | 0.88±0.05^b,B^ | 2.22±0.13^c,D^ | 0.90±0.08^b,C^ | 0.51±0.02^a,B^ |
| Benzyl alcohol | 1894 | 1 | *ND* | *ND* | *ND* | *ND* |
|  |  | 7 | *ND* | *ND* | *ND* | *ND* |
|  |  | 14 | *ND* | *ND* | 5.73±0.17^b,B^ | 5.86±0.16^b,B^ |
|  |  | 21 | *ND* | *ND* | 24.44±0.02^c,C^ | 8.70±0.10^b,C^ |

RI: Retention Index, *ND*: Not Detected.

^a-d^ Values indicated by different letters in the same row are different from each other at *P*<0.05 level.

^A-D^ Values indicated by different letters in the same column are different from each other at *P*<0.05 level.
